# Explaining COVID-19 related mortality disparities in American Indians and Alaska Natives

**DOI:** 10.1038/s41598-023-48260-9

**Published:** 2023-11-28

**Authors:** Wendy S. Slutske, Karen L. Conner, Julie A. Kirsch, Stevens S. Smith, Thomas M. Piasecki, Adrienne L. Johnson, Danielle E. McCarthy, Patricia Nez Henderson, Michael C. Fiore

**Affiliations:** 1 Center for Tobacco Research and Intervention, School of Medicine and Public Health, University of Wisconsin-Madison, Madison, WI USA; 2 Department of Family Medicine and Community Health, School of Medicine and Public Health, University of Wisconsin-Madison, Madison, WI USA; 3 Department of Medicine, School of Medicine and Public Health, University of Wisconsin-Madison, Madison, WI USA; 4 Black Hills Center for American Indian Health, Rapid City, SD USA

**Keywords:** Infectious diseases, Comorbidities, Public health, Risk factors

## Abstract

American Indian and Alaska Native (AI/AN) individuals are more likely to die with COVID-19 than other groups, but there is limited empirical evidence to explain the cause of this inequity. The objective of this study was to determine whether medical comorbidities, area socioeconomic deprivation, or access to treatment can explain the greater COVID-19 related mortality among AI/AN individuals. The design was a retrospective cohort study of harmonized electronic health record data of all inpatients with COVID-19 from 21 United States health systems from February 2020 through January 2022. The mortality of AI/AN inpatients was compared to all Non-Hispanic White (NHW) inpatients and to a matched subsample of NHW inpatients. AI/AN inpatients were more likely to die during their hospitalization (13.2% versus 7.1%; odds ratio [OR] = 1.98, 95% confidence interval [CI] = 1.48, 2.65) than their matched NHW counterparts. After adjusting for comorbidities, area social deprivation, and access to treatment, the association between ethnicity and mortality was substantially reduced (OR 1.59, 95% CI 1.15, 2.22). The significant residual relation between AI/AN versus NHW status and mortality indicate that there are other important unmeasured factors that contribute to this inequity. This will be an important direction for future research.

## Introduction

Health inequities between American Indians and Alaska Natives (AI/AN) and other groups in the United States (US) have persisted throughout the 500 years of colonization^1^. Throughout US history, AI/AN Tribes and tribal communities have been repeatedly and disproportionately impacted by epidemics of diseases such as smallpox and measles^1,2^, and more recently, the H1N1 virus^3^. AI/AN individuals are more likely than non-Hispanic White (NHW) individuals to die from diseases associated with lower respiratory infection such as influenza and pneumonia^4^. Similarly, AI/AN individuals appear to be more severely affected by the COVID-19 pandemic than the general US population. Age-adjusted COVID-19 relative mortality risk of AI/ANs compared to NHW individuals range from 1.8 to 3.8^5–8^. Death from COVID-19 has led to a widening of the life expectancy gap between AI/ANs and NHWs from 7 years in 2019 to 11 years in 2021^9^. Despite declines in COVID-19 mortality from 2021 to 2022, racial and ethnic disparities persist^10^. Provisional 2022 mortality data indicate that COVID-19 was the fourth leading cause of death in the US, and COVID-19 death rates remained high for AI/AN individuals compared to NHW individuals^10^.

Few empirical investigations have explored the potential causes of the disparities in COVID-19 outcomes between AI/AN and NHW individuals. One explanation that has been proposed is that AI/AN individuals experience more medical comorbidities, such as diabetes, liver, and kidney disease than NHW individuals^11,12^, and some of these have been linked to severe COVID-19 outcomes^13,14^. To our knowledge, no study has empirically evaluated whether comorbidities partly explain the COVID-19 mortality disparity for AI/AN individuals. Two studies demonstrated that, at every level of comorbidity, differences between race groups (including AI/AN, Black, and White) persisted, suggesting that factors other than comorbidity contributed to the disparity in COVID-19 mortality^15,16^.

For instance, social and economic conditions in which AI/AN populations live (i.e., area socioeconomic deprivation) may drive inequities in infection and mortality^12^. Historically, US government policies, including forced removal from lands, perpetuated the severity of infectious epidemics by causing starvation, severe crowding, and “historical trauma”^17^. Today, due to these inequities, AI/AN individuals are more likely than NHW individuals to live in neighborhoods characterized by poverty and by housing that is overcrowded and without plumbing^18,19^.

Area socioeconomic deprivation is associated with higher risk for COVID-19 infection^20^, hospitalization and mortality^21^. Wong et al.^19^ examined the extent to which area socioeconomic deprivation contributed to AI/AN disparities in COVID-19 infection in a large geographically diverse sample extracted from the United States Veteran’s Health Administration electronic health records (including 3045 AI/AN US Veterans). The investigators demonstrated that 17–35% of the COVID-19 infection disparity between AI/AN and NHW Veterans could be explained by area socioeconomic deprivation, and this did not differ as a function of whether the Veteran lived on or near a reservation. Studies have not tested the hypothesis that area socioeconomic deprivation explains AI/AN disparities in COVID-19 mortality.

Access to health care is an additional mechanism of disparities in COVID-19 mortality between AI/ANs and NHWs^2,18,22^. AI/ANs often need to travel a great distance to the nearest health care facility and many lack adequate health insurance coverage^18^. To our knowledge, the hypothesis that poorer access to health care may explain disparities between AI/ANs and NHWs in COVID-19 mortality has never been directly tested.

Although most AI/AN individuals (87%) do not reside on reservations or tribal land^23^, most research on socioeconomic deprivation has focused on the 13% minority of AI/AN individuals who do^19^. The experiences of urban versus tribal residing AI/AN individuals differ substantially. For example, urban AI/AN individuals experience less continuity in their health care compared to AI/AN individuals who reside on tribal land^24,25^. There is a critical need for more research on COVID-19 outcomes among urban-dwelling AI/ANs.

The dearth of COVID-19 data on AI/AN individuals in state-level public health surveillance systems has been of grave concern^25–27^. COVID-19 tracking systems have failed to measure or report the race of those affected or to include AI/AN as a distinct category^26,28^. Lack of quality data may be contributing to a widening of health inequities for AI/ANs.

The present study assembled a sample of AI/AN individuals affected with COVID-19 by mining electronic health records (EHRs) of all inpatients affected with COVID-19 from 21 health systems over 2 years. There were 145,944 geographically diverse, predominantly urban patients (87% with known race) hospitalized with COVID-19—546 (0.37%) were AI/AN. The AI/AN inpatients were from 29 different states, which makes this one of the most geographically diverse samples of AI/AN individuals assembled to examine mortality disparities. Importantly, most of the AI/AN patients were living in urban areas, which represents the understudied majority of the AI/AN population^19,29^.

The goals of this study were to (1) explore the potential inequity in COVID-19 mortality in a primarily urban, geographically diverse sample of AI/AN, and to (2) examine whether comorbidities, area social deprivation, and access to treatment might contribute to inequities in mortality. We hypothesized that we would observe the same mortality disparity as found in previous studies of AI/AN and that this disparity would be partially explained by comorbid medical conditions, area social deprivation, and access to treatment.

## Methods

### Study design

The COVID EHR Cohort at the University of Wisconsin (CEC-UW;^30^) is a retrospective cohort study supported by the National Cancer Institute (ClinicalTrials.gov NCT04506528) that included 21 health systems from across the US (see Figure S1 in Supplemental Materials). Data extractions were performed using customized extraction code altered to accommodate unique health system specific EHR features. Each data extraction captured data on new patients meeting inclusion criteria and follow-up data on existing cohort members. Participating health systems provided selected data elements from the EHR of all COVID-19 patients encountered during the study period (February 1, 2020 to January 31, 2022). Data were transferred to the CEC-UW Coordinating Center in Madison, Wisconsin, where they were harmonized and merged. Harmonization, merging, and data analysis occurred September 30, 2021 through July 3, 2023. This study follows the Strengthening the Reporting of Observational Studies in Epidemiology (STROBE) reporting guidelines^31^.

### Ethics statement

The CEC-UW study was initially approved in May 2020 by the University of Wisconsin-Madison Health Sciences Minimal Risk Institutional Review Board (MR-IRB) with approval for the collection of de-identified EHR data from the 21 health systems. The MR-IRB also determined that the study met criteria for a human subjects research exemption and qualified for a waiver of informed consent under the Federal Common Rule. All participating health systems provided written notice of either their own institution’s IRB approval or determination of exemption status before sharing EHR data. In February 2021, the MR-IRB approved a change of protocol for a Limited Data Set, allowing the collection of additional information (e.g., death dates, five-digit zip codes) but excluding direct patient identifiers. Each patient in the data set from each health system was assigned an enduring cryptographically processed Patient ID based on the SHA256 algorithm, which yielded a 64-character unique and private hash-based message authentication code (HMAC).

### Analysis samples

The full CEC-UW inpatient cohort included 145,944 adult patients hospitalized with COVID-19 who had prior contact with the health system and who completed their hospitalization from February 1, 2020 through January 31, 2022 at a participating health system^30^.

This study focused on three analysis samples. One sample comprised all CEC-UW inpatients who identified as AI/AN (N = 546) [78% were non-Hispanic]) or NHW (N = 78,128). The other two were matched samples comprised of all CEC-UW inpatients who identified as AI/AN (N = 546) along with five matched NHW inpatients (N = 2645) selected using the SAS GMATCH macro^32^. (More information is provided below under “Statistical Analysis”).

### Primary outcome

The primary outcome was all-cause in-hospital mortality during the index COVID-19 hospitalization. Because cause of death was not extracted from the EHRs of the participating health systems, we could not definitively attribute it to COVID-19 as some patients could have died during their hospitalization from other causes. Also reported were rates of three severity indicators (admission to the intensive care unit, intubation for ventilator use, and days in the hospital [among patients who did not die prior to their discharge]). The mean age at death was reported among those who died.

### Patient characteristics

The following patient characteristics were extracted from the electronic heath records: sex, age, cigarette smoking status, co-occurring medical conditions (obesity, Type 2 diabetes, kidney, liver, and heart disease, cancer, alcohol use disorder, and drug use disorder [see Supplemental Text S1 for the ICD-10 codes corresponding to the medical conditions]), insurance type, receipt of antiviral medication during the hospitalization (see Supplemental Text S2 for a list of the antiviral medications prescribed), and COVID-19 vaccination status (see Supplemental Text S3). The date of the index hospitalization was extracted to incorporate a variable indicating the year in which the hospitalization occurred. (Note that although it is included as a race category in the EHR, we do not refer to AI/AN as a race based on the recommendation of our Indigenous consultant. The rationale is that AI/ANs are represented by 574 federally recognized tribes that are sovereign political governments and are not a single unified group.)

Additional variables were linked to the EHR data based on the patient’s home ZIP code: region (Northeast, Midwest, South, and West), urbanicity^33^, area social deprivation^34^, and distance to hospital. Urbanicity was based on Rural–Urban Commuting Area (RUCA) codes^33^; codes 1–6 were classified as “urban”, and codes 7–10 were classified as “rural.” Area social deprivation was based on seven census-based indicators of deprivation (see Table S1 in the Supplemental Materials) that were combined via factor analysis^34,35^ (see Supplemental Table S2). The area social deprivation index was calculated at the ZIP code tabulation area level; results are presented for each quintile based on the full sample. The ZIP code of the facility in which treatment was received was used to compute the distance to treatment using a SAS function that calculated the geodetic distance in miles between the centroids of two ZIP code locations, in this case the home and treatment facility ZIP codes. Because distance to treatment was very skewed and kurtotic, it was dichotomized at 60 miles, a distance that represents a significant barrier to getting timely treatment (that is, about 60 min travel time) while still including adequate numbers in each group for analyses.

These variables were included as covariates in adjusted multivariable models (covariate categorizations are given in Table 2).

### Statistical analysis

Matching was conducted to create samples that were aligned on key variables while allowing for variability in important predictors. Two different matched samples were created. One set of matched samples (Match 1) was matched on age, region, month/year, and sex. The other set of matched samples (Match 2) was matched on region, month/year, and sex (not age). Because age was confounded with AI/AN versus NHW status in the full sample (see Table 2), NHW patients were matched to the AI/AN patients based on age (± 5 years) in Match 1. AI/AN and NHW patients were not matched on age when the outcome was age at death because age at admission and age at death for those who died during their hospitalization were nearly perfectly correlated (Match 2). Matching on region of residence and time of hospitalization minimized potential differences in availability of SARS CoV-2 vaccinations or exposure to different COVID-19 strains for AI/AN and NHW (Match 1 and 2). Both samples were matched based on sex (Match 1 and 2) to maintain the nearly equal proportions of men and women.

Analyses were conducted in the matched samples to identify factors that may contribute to disparities in mortality. First, we examined whether there were differences between AI/AN and NHW inpatients for each of the patient characteristics to identify potential explanations for the inequities in mortality. Second, we examined the extent to which each of these patient characteristics predicted mortality individually and after controlling for all the other characteristics in adjusted models. Of particular interest was the extent to which the association between AI/AN versus NHW status and mortality were reduced after patient characteristics, such as smoking and medical comorbidities, access to treatment (as indicated by distance to treatment, insurance type, residence in a rural or urban region, and receipt of antiviral medication while hospitalized), and area social deprivation were considered. The impact of controlling for these characteristics was quantified by examining the percentage attenuation in the effect sizes obtained in unadjusted and adjusted models: 100 × (B_unadjusted model_ − B_adjusted model)_/(B_unadjusted model_) cf.^36,37^. Third, in the fully adjusted models, we examined whether any of the patient characteristics differentially predicted mortality in AI/AN and NHW inpatients. The purpose was to determine whether a potential risk factor exerted a greater or lesser effect on mortality among AI/AN compared to NHW individuals. Analyses were conducted in SPSS^38^. Multilevel generalized linear models with a binomial distribution and a logit link were fit. Multilevel analysis was used to account for the clustering of patients within the 21 health systems.

Bootstrapped confidence intervals around percentages and means and t-tests were estimated in SPSS. The Benjamini–Hochberg procedure^39^ was applied to control the false discovery rate in the multivariable analyses and results of such correction are shown in relevant tables.

## Results

### Differences in mortality and other outcomes between AI/AN and NHW inpatients

**Figure 1:**
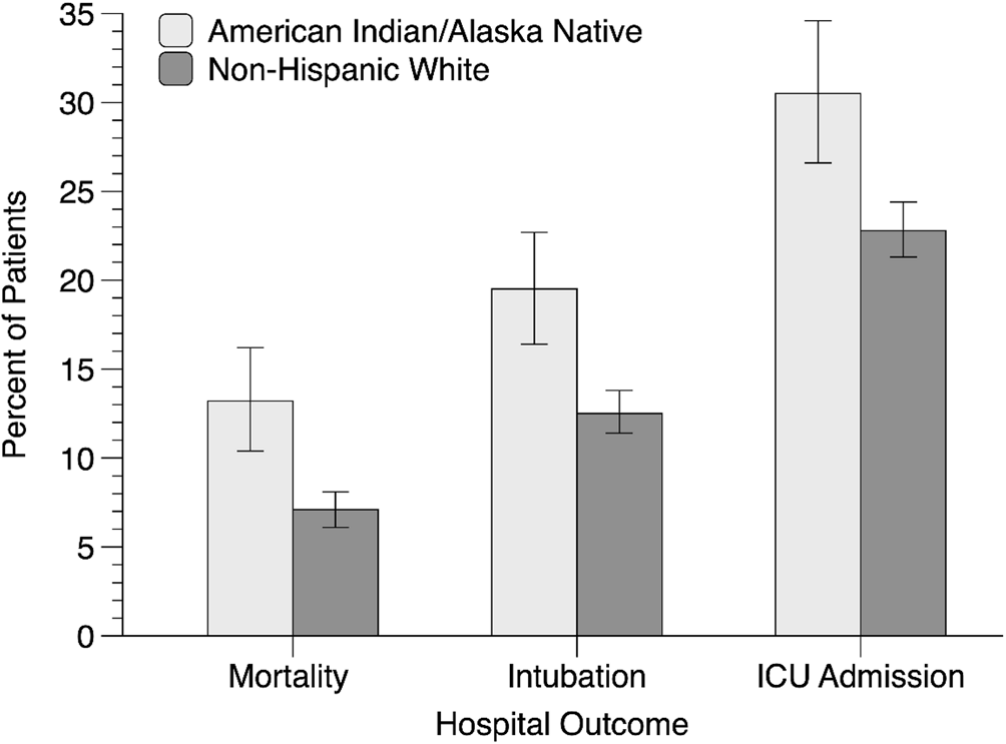
In-hospital mortality, intubation, and ICU admission among matched samples of American Indian/Alaska Native and Non-Hispanic White inpatients from the CEC-UW COVID-19 cohort. Vertical bars are bootstrapped 95% confidence intervals, analyses are based on the Match 1 sample.

AI/AN patients were more likely to die during their hospitalization than were NHW patients (Table 1 and Fig. 1). AI/AN patients died at a significantly younger age than NHW patients in the full and matched samples, which was not surprising given that AI/ANs were admitted at a younger age. AI/ANs were also more likely to require intubation, be admitted to the intensive care unit, and spend more days in the hospital than NHWs; Mann–Whitney tests indicated that AI/ANs and NHWs did not significantly differ in the number of days hospitalized in the full sample but did in the matched sample (Table 1).

**Table 1:** Comparisons between American Indian/Alaska Native and Non-Hispanic White adult inpatients from the CEC-UW COVID-19 cohort on in-hospital mortality and other outcomes.

	American Indian/Alaska Native	Non-Hispanic White		Full sample comparison			Matched sample comparison		
		Full sample	Matched sample						
	(a)	(b)	(c)	(a versus b)			(a versus c)		
Mortality									
				χ^2^ *df* = 1	*p*	OR	χ^2^ *df* = 1	*p*	OR
In-hospital death^a^									
%	13.2	9.0	7.1	11.30	0.001	1.54	22.62	< .0001	1.98
Other outcomes									
				*U*	*p*	*z*	*U*	*p*	z
Age at death^b^									
Mean	64.50	73.87	73.66	438,634.5	< .001	−6.8	14,931.5	< .001	−6.3
SD	15.64	12.64	12.54						
				χ^2^ *df* = 1	*p*	OR	χ^2^ *df* = 1	*p*	OR
Intubation^a^									
%	19.6	12.4	12.5	25.64	< .001	1.72	16.94	< .0001	1.72
ICU admission^a^									
%	30.6	20.2	22.8	36.52	< .001	1.75	13.56	0.0002	1.50
				*U*	*p*	*z*	*U*	*p*	z
Days hospitalized^a^									
mdn	5.02	4.93	4.70	17,479,793	0.16	1.4	619,171.5	0.02	2.34

*ICU* intensive care unit, *df* degrees of freedom, *SD* standard deviation, *OR* odds ratio, *mdn* median.

^a^Match 1 sample used for matched analyses.

^b^Match 2 sample used for matched analyses.

### Differences in patient characteristics between AI/AN and NHW inpatients

Differences in patient characteristics between AI/ANs and the full and matched NHW samples are presented in Table 2. As mentioned, AI/AN patients were significantly younger than the full NHW sample but, as expected, were not younger than the matched NHW sample. Some of the significant differences observed in the full sample (smoking status, obesity, heart disease, drug use disorder, insurance type, and vaccination status) were no longer significant in the matched samples, and one new difference (chronic renal failure) was revealed by the matching process.

**Table 2:** Comparisons between American Indian/Alaska Native and Non-Hispanic White adult inpatients from the CEC-UW COVID-19 cohort on comorbid disorders, treatment access, and area deprivation.

Characteristic	American Indian/ Alaska Native N = 546		Non-Hispanic White					*p* value^b^
			Full sample N = 78,128		*p* value^a^	Matched sample^d^ N = 2,645		
	N	%	N	%		N	%	
Sex					0.698			0.948
Female	270	49.5	37,984	48.6		1299	49.1	
Male	276	50.5	40,412	51.4		1346	50.9	
Age					< 0.001			0.833
18–49	188	34.4	14,647	18.7		874	33.0	
50–64	164	30.0	19,737	25.3		805	30.4	
65+	194	35.5	43,744	56.0		966	36.5	
Smoking status					0.038			0.95
Never smoker	288	52.7	38,441	49.2		1402	53.0	
Former smoker	178	32.6	28,989	37.1		877	33.2	
Current smoker	55	10.1	6252	8.0		256	6.7	
Missing	25	4.6	4446	5.7		110	4.2	
Current comorbidities								
Obesity	159	29.1	19,337	24.8	0.018	695	26.3	0.184
Diabetes (Type 2)	224	41.0	21,449	27.5	< 0.001	640	24.2	< 0.001
Chronic renal failure	113	20.7	15,669	20.1	0.231	444	16.8	0.026
Liver disease	64	11.7	4807	6.2	< 0.001	173	6.5	< 0.001
Heart disease	331	60.6	50,853	65.1	0.029	1493	56.5	0.070
Cancer	41	7.5	6690	8.6	0.380	207	7.8	0.818
Alcohol use disorder	39	7.1	2416	3.1	< 0.001	104	3.3	0.001
Drug use disorder	27	4.9	2245	2.9	0.004	110	4.2	0.399
Insurance type					< 0.001			< 0.001
Commercial	142	26.0	21,190	27.1		1014	38.3	
Medicare	251	46.0	46,960	60.1		1172	44.3	
Medicaid	98	17.9	4677	6.0		261	9.9	
Uninsured	20	3.7	1289	1.6		51	1.9	
Other	35	6.4	4012	5.1		147	5.7	
Received antiviral medication					0.880			0.498
No	270	49.6	39,042	50.0		1355	51.2	
Yes	274	50.4	39,086	50.0		1290	48.8	
Distance to treatment					< 0.001			< 0.001
< 60 miles	420	76.9	73,246	93.8		2589	90.3	
≥ 60 miles	126	23.1	7898	6.2		256	9.7	
Region					< 0.001			0.860
Northeast	109	20.0	26,714	34.2		540	20.4	
Midwest	145	26.6	27,102	34.7		725	27.4	
South	132	24.2	20,367	26.1		660	25.0	
West	160	29.3	3909	5.0		720	27.2	
Urbanicity					< 0.001			< 0.001
Urban	472	86.4	72,727	93.1		2465	93.2	
Rural	74	13.6	5360	6.9		180	6.8	
Area social deprivation quintile					< 0.001			< 0.001
Lowest 20%	38	7.1	15,328	19.8		518	19.9	
20–40%	48	9.0	15,602	20.1		459	17.7	
40–60%	96	17.9	15,586	20.1		522	20.1	
60–80%	94	17.6	15,471	20.0		525	20.2	
Highest 20%	259	48.4	15,514	20.0		576	22.2	
Year					0.062			0.530
Feb 2020–Dec 2020	231	42.3	30,007	38.4		1075	40.6	
Jan 2021–Jan 2022	315	57.7	48,121	61.6		1570	59.4	
Fully vaccinated^c^	181	33.2	30,265	38.7	0.026	993	37.5	0.663
No	479	88.1	66,140	84.7		2311	87.4	
Yes	65	11.9	11,988	15.3		334	12.6	

^a^Chi-square tests comparing proportions to AIAN.

^b^Cochran–Mantel–Haenszel tests comparing proportions to AIAN.

^c^Fully vaccinated before hospitalization; see Supplemental Text S2.

^d^Match 1 sample used for matched analyses; samples were matched on sex, age, region, and year.

In the matched samples, AI/AN patients who were hospitalized with COVID-19 were significantly more likely than NHW patients to be diagnosed with the comorbid conditions of Type 2 diabetes, chronic renal failure, liver disease, and alcohol use disorder. They also had significantly higher comorbidity burden as indicated by a count of the number of comorbid disorders (median 2.0) than NHW patients (median 1.0); Mann–Whitney test (*U* = 842,344.00, *z* = 6.47, *p* < 0.001).

Although the AI/AN patients were predominantly urban dwelling (86.4%), they were more likely than NHW patients to live in a rural area and to live 60 or more miles from the facility in which they received treatment. AI/AN patients were significantly more likely than NHW patients to live in socioeconomically deprived areas. For example, they were more likely to live in areas with a higher percentage of residents living below the federal poverty level, with less than a high school education, who did not own a car, who lived in a crowded housing unit and in households headed by a single parent (see Supplemental Table S1). In summary, these results suggest that comorbid medical conditions, access to treatment, and area social deprivation are potential contributors to the inequity in mortality between AI/AN and NHW individuals with COVID-19. AI/AN and NHW patients did not significantly differ in smoking status, some medical comorbidities (obesity, heart disease, cancer, and drug use disorder), type of insurance coverage, receipt of antiviral medication, year admitted to the hospital, and vaccination status. These results suggest that these are unlikely to be contributors to the inequity in mortality between AI/AN and NHW individuals with COVID-19.

### Associations of AI/AN versus NHW status and patient characteristics with mortality

As presented in Table 3, several comorbidities were associated with in-hospital mortality (after covariate adjustment and control for false discovery rate): chronic renal failure, liver disease, and heart disease. Several indicators of health care access were associated with in-hospital mortality in unadjusted analyses: being a Medicare recipient, receipt of antiviral medication, living a greater distance from the treatment facility, and living in a rural area; being in the top quintile of area social deprivation was also associated with in-hospital mortality, but after covariate adjustment and control for false discovery rate, being a Medicare recipient and receipt of antiviral medication were the only ones that remained significant predictors of mortality.

**Table 3:** Associations of comorbid disorders, treatment access, and area deprivation with mortality among matched samples^a^ of American Indian/Alaska Native and Non-Hispanic White adult inpatients from the CEC-UW COVID-19 cohort.

Patient Characteristic	Unadjusted associations		Adjusted associations^b^		
	OR	95% CI	OR	95% CI	*B-H?*
Group					
Non-Hispanic White (ref)	–	–	–	–	
American Indian/Alaska Native	1.98	1.48, 2.65	1.59	1.15, 2.22	Yes
*Sex (matching variable)*					
*Age (matching variable)*					
Smoking status					
Never smoker (ref)	–	–	–	–-	
Former smoker	1.26	0.96, 1.65	0.94	0.70, 1.26	No
Current smoker	0.51	0.29, 0.92	0.47	0.25, 0.88	No
Missing	1.37	0.76, 2.47	1.69	0.90, 3.19	No
Current comorbidities					
Obesity	1.51	1.15, 1.98	1.36	1.01, 1.84	No
Diabetes (Type 2)	1.75	1.34, 2.28	0.89	0.66, 1.20	No
Chronic renal failure	2.38	1.80, 3.15	1.70	1.24, 2.32	Yes
Liver disease	2.77	1.93, 3.99	2.64	1.74, 4.01	Yes
Heart disease	4.52	3.20, 6.38	3.47	2.38, 5.06	Yes
Cancer	1.88	1.27, 2.79	1.69	1.11, 2.59	No
Alcohol use disorder	0.77	0.39, 1.53	0.76	0.35, 1.63	No
Drug use disorder	0.70	0.34, 1.45	1.23	0.55, 2.75	No
Insurance type					
Commercial (ref)	–	–	–	–	
Medicare	3.42	2.45, 4.77	2.52	1.73, 3.66	Yes
Medicaid	1.05	0.58, 1.91	0.94	0.49, 1.81	No
Uninsured	1.40	0.49, 4.03	1.63	0.54, 4.91	No
Other	2.07	1.13, 3.81	2.11	1.11, 4.01	No
Received antiviral medication					
No (ref)	–	–	–	–	
Yes	1.91	1.46, 2.50	1.65	1.24, 2.19	Yes
Distance to treatment					
< 60 miles (ref)	–	–	–	–	
≥ 60 miles	1.67	1.18, 2.37	1.17	0.76, 1.79	No
*Region (matching variable)*					
Urbanicity					
Urban (ref)	–	–	–	–	
Rural	1.66	1.10, 2.52	1.20	0.74, 1.96	No
Area social deprivation quintile					
Lowest 20% (ref)	–	–	–	–	
20–40%	1.20	0.74, 1.94	0.98	0.59, 1.63	No
40–60%	1.11	0.69, 1.77	0.99	0.61, 1.62	No
60–80%	1.36	0.86, 2.14	1.17	0.73, 1.89	No
Highest 20%	1.80	1.19, 2.74	1.53	0.97, 2.40	No
*Year (matching variable)*					
Fully vaccinated					
No (ref)	–	–	–	–	
Yes	0.71	0.46, 1.09	0.61	0.39, 0.97	No

*OR* odds ratio, *CI* confidence interval, *B-H?*  did the *p* value survive Benjamini & Hochberg (1995) test for multiple comparisons. Italic rows indicate variables on which groups were matched.

^a^Match 1 sample used for all analyses.

^b^Adjusted analyses estimate the effect of each characteristic after adjusting for the influence of every other characteristic.

The unadjusted odds ratio of the association between AI/AN versus NHW status and mortality was 1.98 (95% CI 1.48, 2.65). After adjusting for comorbidities, access to treatment, area social deprivation and vaccination status, the odds ratio was reduced to 1.59 (95% CI 1.15, 2.22), which represents a 32% reduction in the association between AI/AN versus NHW status and mortality.

Analyses were conducted to identify the source of this diminution in the association between AI/AN versus NHW status and mortality. In a model that adjusted for only comorbidities (excluding those that were inversely associated with mortality in the adjusted model), the odds ratio was reduced to 1.82 (95% CI 1.35, 2.46), which represents a 12% reduction in the association between AI/AN versus NHW status and mortality. This effect was further probed by individually examining each of the six comorbid disorders. The single disorder that accounted for most of the mortality disparity was any liver disease (odds ratio reduced to 1.87 [95% CI 1.40, 2.51], representing a 9% reduction; the others ranged from 1.92 to 1.99). When specific liver diseases were included in the adjusted model presented in Table 3, the single best predictor of mortality with COVID-19 was hepatic failure, not elsewhere classified (OR 8.05, 95% CI 4.22, 15.36). Given that liver disease is often secondary to hepatitis B and C and HIV infections^40^, we conducted post hoc analyses to determine that the relation between liver disease and mortality could not be explained by comorbid hepatitis or HIV (see Supplemental Text S4).

In a model that adjusted for only access to treatment (distance to treatment, insurance type, residence in a rural or urban region, and receipt of antiviral medication while hospitalized), the odds ratio was reduced to 1.82 (95% CI 1.34, 2.47), also representing a 12% reduction. In a model that adjusted for only area social deprivation, the odds ratio was reduced to 1.79 (95% CI 1.32, 2.43), representing a 15% reduction. These results suggest that comorbidities (especially comorbid liver disease), access to treatment and area social deprivation all contribute to the disparity in COVID-19 related mortality between AI/AN and NHW individuals. The significant residual relation between AI/AN versus NHW status and mortality after accounting for these candidate explanatory variables indicate that they do not fully account for this disparity.

### Moderation of associations between AI/AN versus NHW status and mortality

We examined whether any of the patient characteristics differentially predicted mortality in AI/AN and NHW inpatients in fully adjusted models by including an interaction term between AIAN versus NHW status and each of the patient characteristics. After control for false discovery rate, there was no evidence that any of the potential risk factors exerted a greater or lesser effect on mortality among AI/AN compared to NHW individuals (see Supplemental Table S3). Before control for false discovery rate, however, there was a significant interaction between AIAN versus NHW status and alcohol use disorder (see Supplemental Text S5). The magnitude of the association between AI/AN versus NHW status and mortality did not change from 2020 to 2022.

## Discussion

This study compared the outcomes of 546 AI/AN to 78,128 NHW individuals from 21 US health systems who were hospitalized with COVID-19 from February 1, 2020 to January 31, 2022. To better isolate differences, the AI/AN sample was also compared to a sample of 2645 NHW individuals matched on age, sex, region, and month/year of hospital admission. As expected, based on previous epidemics and recent studies^4–7^, the odds of in-hospital mortality were doubled in the AI/AN compared to the matched sample of NHW individuals with COVID-19. Novel to this study was the direct examination of the extent to which comorbidities, area social deprivation, and access to treatment could contribute to the AI/AN disparity in mortality.

### Comorbidity and mortality differences between AI/AN and NHW

AI/AN inpatients were compared to NHW inpatients on eight diseases known to be more common among AI/AN^12,25^ and that have been linked to severe COVID-19 outcomes^13,14^. In this inpatient sample, AI/AN patients were more likely to have been diagnosed with three of these (diabetes, chronic renal failure, and liver disease); liver disease accounted for the largest portion of the comorbidity-related mortality disparity between AI/AN and NHW patients.

### Area social deprivation and mortality differences between AI/AN and NHW

AI/AN inpatients were more than twice as likely to reside in the most socially deprived areas relative to NHW inpatients. Consistent with prior research^19,41^ higher area social deprivation increased the risk of COVID-19 inpatients’ dying in the hospital. Furthermore, adjusting for area social deprivation attenuated the association between AI/AN versus NHW status and hospital mortality. The present findings extend the limited number of studies that have linked area social deprivation with patient-level COVID-19 clinical outcomes.

### Access to treatment and mortality differences between AI/AN and NHW

Higher rates of intubation, ICU admission, and days hospitalized suggest that AI/AN inpatients may have presented to inpatient care later in the COVID-19 disease course compared to NHW inpatients. In this study, 23% of AI/AN inpatients lived 60 or more miles from their treatment facility, compared to only 10% of the matched sample of NHW. It is likely that this greater distance to treatment may account for the greater severity of COVID-19 illness and ultimately higher rates of death in AI/AN than NHW patients. The association between AI/AN versus NHW status and mortality was reduced after adjusting for distance to treatment. More research is needed to identify other obstacles to timely treatment that AI/AN individuals are more likely to encounter than their NHW counterparts^42^. In particular, transportation barriers^43^ and lack of internet access^17,18^ may also impede timely treatment.

### Differences between AI/AN and NHW in age at death

Previous studies have documented younger ages at death for AI/AN than NHW individuals with COVID-19^9,44^. In the full sample of the present study, AI/AN patients were younger (*d* = 0.74) and had younger in-hospital ages at death (*d* = 0.74) than NHW patients. Of those who died during their hospitalization, 60% of the AI/ANs compared to only 34% of the NHWs were less than 70 years old. The contributors to mortality disparities previously discussed also likely contributed to disparities in age at death. AI/ANs were more likely than NHWs to be living in crowded housing, which may have promoted greater viral load. Living further from treatment may have led to greater delays in getting or seeking treatment and more advanced illness at hospitalization for AI/ANs compared to NHWs.

Disparities in age at death might also be explained by the concept of “weathering” that posits that the cumulative impact of repeated experience with social or economic adversity and political marginalization may lead to physiological deterioration^45^. This cumulative wear and tear has been termed “allostatic load”^45^. An empirical demonstration of weathering was conducted in a community-based sample of Black and White individuals showing that the allostatic load of a 40-year-old Black person was equal to that of a 50-year-old White person^46^. To our knowledge, studies of the causes of disparities in age at death have not been conducted among AI/AN individuals but should be a top priority for future research. The profound loss of lifespan among AI/AN has widespread effects given the value that Indigenous communities place on their elders^28,47^.

### Other studies quantitatively explaining COVID-19 related mortality disparities

Several studies have systematically documented COVID-19 related mortality disparities among minority populations (e.g.,^48,49^). To our knowledge, only two studies, both conducted in the UK, have attempted to quantitatively explain the potential causes of these mortality disparities^36,50^. In one UK study^36^, comorbidities explained 10% of the association between Black/White status and mortality and 39% of association between South Asian/White status and mortality; social factors (educational attainment, occupational attainment, household size and area deprivation) explained 28% of the association between Black/White status and mortality and 4% of association between South Asian/White status and mortality. In the other UK study^50^, comorbidities and social factors (household size and area deprivation), but not lifestyle factors (smoking, body mass index), explained about 40% of the association between Black/White status and mortality and between South Asian/White status and mortality. (Access to treatment was not included in either of the UK studies.) In the present study, comorbidities explained 12% of the association between AI/AN status and mortality and area social deprivation explained 15% of the association between AI/AN status and mortality. Taken together, results from the UK studies and the present study suggest that comorbidities and area social deprivation account for similarly small fractions of the COVID-19 related mortality disparity among Black, South Asian, and AI/AN individuals. Much of the disparity was left unexplained for all three ethnic groups.

### Limitations

First, the sample included only hospitalized AI/AN and NHW patients during their first hospitalization for COVID-19, so it does not reflect the course of COVID-19 and mortality differences in the broader AI/AN population. Second, outcomes occurring post-discharge or outcomes occurring at nonparticipating health systems were not captured. Third, results across time could not be linked with type of COVID-19 variant. The analyses were conducted over the first 2 years of the pandemic, suggesting that the data obtained were contemporaneous with high prevalence of alpha, delta, and early omicron variants^51^. Fourth, risk factors for COVID-19 related mortality were considered in isolation when they were more likely to act in concert or be stages in a causal chain. For example, area social deprivation and distance to treatment may be barriers to adequate prevention and intervention for comorbid liver disease^52^. Future research should model the process by which risk factors combine to influence COVID-19 related mortality.

Fifth, the CEC-UW inpatient cohort is not a representative sample. There was selection of the participants based on having a diagnosis of COVID-19 and for being hospitalized. It is well known that studies based on EHR data are plagued by collider bias^53–55^. In this case, COVID-19 may be a collider associated with both AI/AN versus NHW status and mortality and could have induced distorted or spurious findings^56^. It is reassuring that the association between AI/AN versus NHW status and mortality observed in this study was similar to results obtained from other sources, such as state-level surveillance systems^5,7,8^. However, the positive association of receipt of antiviral medication with mortality may have been due to collider bias (but also possibly due to sicker patients being more likely to be prescribed medication). Although the association with mortality may have been distorted, it was an important observation that AI/AN were not less likely than NHW to receive pharmacologic treatment for their COVID-19.

## Conclusions

Notwithstanding limitations, this study is an important contribution to the literature because it represents the first attempt to explain COVID-19 related mortality disparities among AI/AN. Comorbidities, area social deprivation, and access to treatment were all important contributors to the mortality disparity between AI/AN and NHW inpatients with COVID-19. Nonetheless, the significant residual relation between AI/AN versus NHW status and mortality after accounting for the candidate explanatory variables of comorbidity burden, neighborhood socioeconomic deprivation and reduced access to health care indicate that there are other important unmeasured factors that contribute to this inequity. This likely includes living conditions, such as multigenerational and crowded housing^57^, being a frontline worker^57^, and having inadequate access to transportation^43^ and to the internet^17,18^. Accounting for the unexplained causes of disparities among AI/AN will be an important direction for future research.

Health disparities among AI/AN are not a new problem but reflect “legacies of failing to address historical and ongoing inequities” (^58^, p. 2739). Results of the present study likely extend beyond the current COVID-19 pandemic and may apply to many other past, current, and future health disparities experienced by AI/AN. The availability of quality data on the disparate impacts of health threats such as COVID-19 on AI/ANs is essential in the effort to reduce disparities and enhance health equity.

### Supplementary Information

Supplementary Information.

## Data Availability

The existing Data Transfer and Use Agreements negotiated with each of the participating health systems preclude the University of Wisconsin from sharing CEC-UW data with any entity. Information Management Services, Inc. (IMS), under contract with the National Cancer Institute, is responsible for housing the CEC-UW dataset. Investigators desiring access to CEC-UW data can apply to IMS (https://www.imsweb.com/).
