# Supplementary material for: Explaining COVID-19 related mortality disparities in American Indians and Alaska Natives
Source: Sci Rep. 2023 Nov 28;13:20974. doi: 10.1038/s41598-023-48260-9 (PMC10684501; doi:10.1038/s41598-023-48260-9)
Supplement: Supplementary file 1 — Supplementary Information. [file 41598_2023_48260_MOESM1_ESM.pdf]

Supplemental Materials

for

Explaining COVID-19 Related Mortality Disparities in American Indians and Alaska Natives

Contents

Text S1.	ICD-10 codes of comorbid medical conditions.
Text S2.	Antiviral medications prescribed.
Text S3.	Vaccination status.
Text S4.	Post hoc analyses examining whether hepatitis B or C or HIV explain the association between liver disease and mortality.
Text S5.	Probing the significant interaction between AI/AN versus NHW status and alcohol use disorder in predicting mortality.
Table S1.	Matched sample analyses of differences in specific indicators of area social disadvantage among CEC-UW American Indian/Alaska Native and Non-Hispanic White inpatients with COVID-19.
Table S2	Factor loadings of Social Deprivation Index Items
Table S3.	Tests of interactions between AI/AN versus NHW status and patient characteristics on mortality.
Figure S1.	CEC-UW Participating health systems.

Text S1. ICD-10 codes of comorbid medical conditions

ICD-10 codes for the following medical conditions were extracted from the admission, encounter, and discharge diagnosis fields in the electronic health record and were combined into a composite diagnosis when necessary.

Comorbid disorder	ICD-10 codes
Obesity	E66.0, E66.01, E66.09, E66.1, E66.2, E66.8, E66.9
Diabetes (Type 2)	E11.x
Chronic renal failure	N18.x, Z99.2
Liver disease	K70 – K77
Heart disease	I4, I5A, I10, I15, I16, I20 – I28, I50-I52
Cancer	C00 – C95
Alcohol use disorder	F10.1 - F10.2
Drug use disorder	F11.1 – F11.2, F12.1 – F12.2, F13.1 – F13.2, F14.1 – F14.2, F15.1 – F15.2, F16.1 – F16.2, F18.1 – F18.2, F19.1 – F19.2
Hepatitis B and C	B16-B19
HIV	B20

Text S2. Antiviral medications prescribed during hospitalization.

Medication
Remdesivir
Tocilizumab
Baricitinib
Casirivimab
Imdevimab
Casirivimab
Bamlanivimab
Etesevimab
Bamlanivimab + Etesevimab
Sarilumab
Molnupiravir
Nirmatrelvir
Ritonavir
Lopinavir
Combination of Nirmatrelvir + Ritonavir (aka Paxlovid)
Sotrovimab
Cilgavimab
Tixagevimab
Cilgavimab + Tixagevimab (aka Evusheld)

Text S3. Vaccination status.

Records indicating date and occurrence of up to three COVID-19 vaccinations were extracted from the EHRs. This information was combined to yield a variable indicating whether the patient had been fully vaccinated prior to the index inpatient hospitalization. This variable did not consider whether the patient was hospitalized on or after the availability of vaccinations on December 11, 2020 or the maker of the vaccination(s).

As reported in Table 2, the numbers of AI/AN and NHW participants who were fully vaccinated prior to their admission to the hospital were 65 (11.9%) and 11,988 (15.3%), respectively. The numbers of AI/AN and NHW participants who were hospitalized on or after the availability of vaccinations was 358/546 = 65.6% and 54,249/78,128 = 69.4%, respectively; the percentage of AI/AN and NHW participants who were fully vaccinated among these subsamples were 18.2 and 22.1%, respectively ($\chi^2(1) = 3.21$, $p = 0.07$).

Of the 12,063 inpatients who had received at least two doses (or one dose of Janssen) prior to their visit, 6,698 (55.5%) had received two doses of Pfizer, 3,974 (32.9%) had received two doses of Moderna, 1,289 (10.69%) had received at least one dose of Janssen, and the remaining 102 (< 1%) patients had received two doses of Astrazeneca or Novavax or a combination of Pfizer, Moderna, Janssen or an unspecified vaccine.

There is no indicator of missingness of the vaccination variables. Vaccinations that occurred outside of the health system (i.e., at pharmacies or retailers) may not have been transmitted to the health care system at which the index hospitalization occurred.

Text S4. Post hoc analyses examining whether hepatitis B or C or HIV explain the association between liver disease and mortality.

ICD-10 codes corresponding to Hepatitis B or C and HIV are in Supplemental Text S1.

Hepatitis B or C

There were 14 (2.6%) and 44 (1.7%) AI/AN and NHW inpatients, respectively, who had a current diagnosis of hepatitis B or C in their EHR, and this did not significantly differ (χ^2 = 2.09, df=1, p = .148). Before including hepatitis B or C in the model, the association between any liver disease and mortality was OR = 2.64 (95% CI = 1.74, 4.01). After including hepatitis B or C in the model, the association between any liver disease and mortality was OR = 2.81 (95% CI = 1.84, 4.28).

HIV

There were 3 (0.6%) and 8 (0.3%) AI/AN and NHW inpatients, respectively, who had a current diagnosis of HIV in their EHR, and this did not significantly differ (χ^2 = 0.81, df=1, p = .367). There were too few inpatients with HIV to include this in models predicting mortality.

In sum, it does not appear that the risk of mortality associated with liver disease is explained by hepatitis infection or HIV.

Text S4. Probing the significant interaction between AI/AN versus NHW status and alcohol use disorder in predicting mortality.

To probe the significant interaction between AI/AN versus NHW status and alcohol use disorder in predicting mortality we re-ran full models separately for AI/AN and NHW.
The adjusted odds ratios of the associations were:
AI/AN: aOR = 1.51 (95% CI = 0.54, 4.22)
NHW: aOR = 0.13 (95% CI = 0.02, 0.99)

To remove the potential influence of comorbid disorders, we also conducted univariable analyses.
The unadjusted odds ratios were:
AI/AN: OR = 1.77 (95% CI = 0.78, 4.03)
NHW: OR = 0.12 (95% CI = 0.02, 0.90)

Note that the interaction that motivated these stratified analyses did not survive Bonferroni or False Discovery Rate corrections. Nonetheless, these results seem to suggest that a diagnosis of alcohol use disorder in the EHR of NHW individuals hospitalized with COVID-19 was associated with a significantly lower odds of mortality, whereas a diagnosis of alcohol use disorder in the EHR of AI/AN individuals hospitalized with COVID-19 was associated with a non-significantly higher odds of mortality.

Table S1. Matched sample analyses of differences in specific indicators of area social disadvantage among CEC-UW American Indian/Alaska Native and Non-Hispanic White inpatients with COVID-19

	Race Group					
**Characteristic**	**American Indian/ Alaska Native** N = 536		**Non-Hispanic White** N = 2,602		**Difference between AIAN and NHW**	
**Characteristics at the zip-code level**	**Mean**	**SD**	**Mean**	**SD**	***d***	**95% CI**
Social Deprivation Index	60.75	29.03	42.15	28.51	0.65	0.57, 0.75
Percent population…						
…less than 100% federal poverty level	19.41	11.35	13.01	8.32	0.72	0.63, 0.82
…single-parent households	20.97	8.92	16.05	7.23	0.66	0.56, 0.75
…age 25+ with <12 years of education	16.05	10.32	11.06	7.40	0.63	0.53, 0.72
…with no car	13.78	15.76	8.91	12.24	0.38	0.29, 0.47
…living in renter occupied housing units	40.13	19.29	32.48	17.28	0.44	0.34, 0.53
…living in crowded housing units	5.05	5.90	2.79	3.23	0.59	0.50, 0.69
…non-employed (not seeking work)	10.02	6.42	7.94	4.08	0.46	0.37, 0.55

Note: *d, or Cohen's d,* is an effect size estimate that is calculated as the standardized mean difference between two groups.

Table S2. Factor loadings of the specific indicators of area social disadvantage obtained at the Zip Code Tabulation Area (ZCTA) level

**Characteristic**	**Factor loading**
Percent population…	
…less than 100% federal poverty level	0.90
…single-parent households	0.75
…age 25+ with <12 years of education	0.79
…with no car	0.69
…living in renter occupied housing units	0.72
…living in crowded housing units	0.61
…non-employed (not seeking work)	0.57

Data Source: Analyses of American Community Survey (ACS), 2015-2019 5-Year Summary Files; obtained from https://www.graham-center.org/maps-data-tools/social-deprivation-index.html

Table S3. Tests of interactions between AI/AN versus NHW status and patient characteristics on mortality.

**Patient Characteristic**	Mortality		
	F	*df*	*p*
Sex			
Age group			
Smoking status	2.268	3, 3106	0.079
Obesity	0.927	1, 3108	0.336
Diabetes (Type 2)	1.863	1, 3108	0.172
Chronic renal failure	0.024	1, 3108	0.877
Liver disease	1.934	1, 3108	0.164
Heart disease	1.812	1, 3108	0.178
Cancer	1.757	1, 3108	0.185
Alcohol use disorder	5.666	1, 3108	0.017
Drug use disorder	0.389	1, 3108	0.533
Insurance Status	1.202	4, 3105	0.308
Received antiviral medication	2.323	1, 3108	0.128
Distance to Treatment	2.676	1, 3108	0.102
Region			
Urbanicity	0.646	1, 3108	0.422
Area Social Deprivation	1.529	4, 3105	0.191
Year[a]	0.012	1, 78002	0.912
Vaccination	0.530	1, 3108	0.467

Note: None of the interactions were significant after applying a false discovery rate correction. Gray cells indicate variables on which groups were matched.

[a] This analysis was conducted in the full sample because month/year of the index hospitalization was included in the derivation of the matched samples.

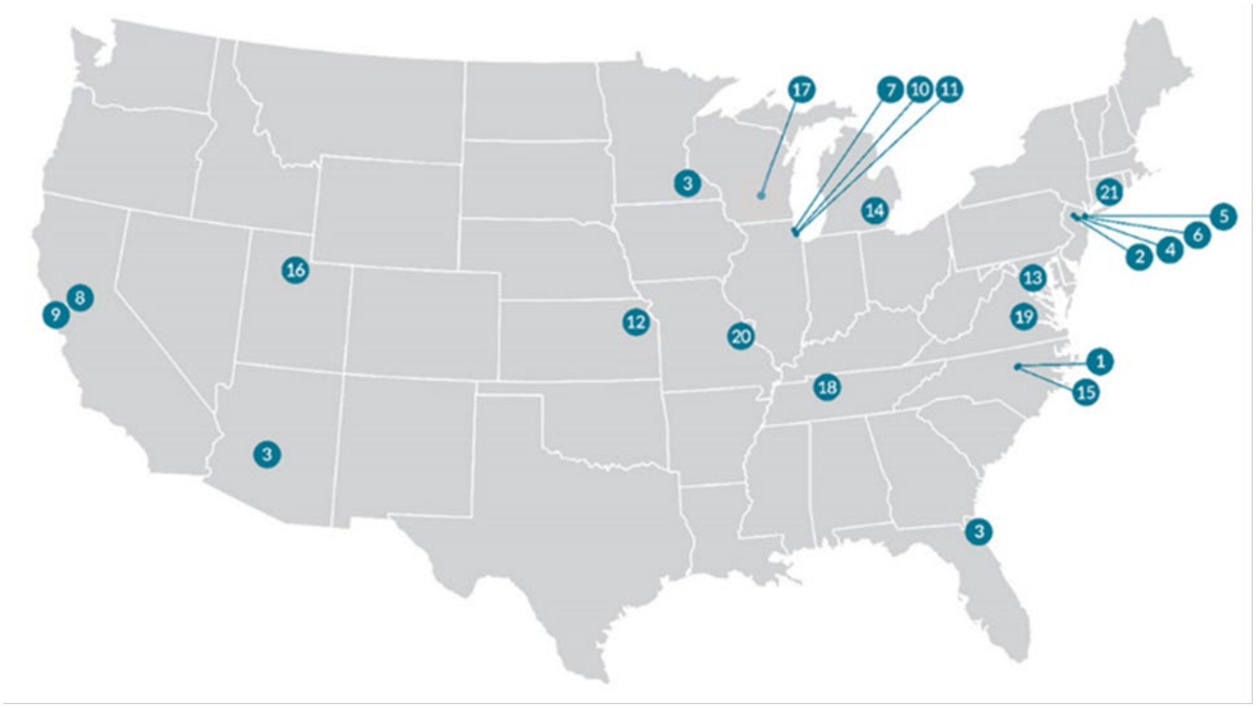

1. Duke University	2. Hackensack Meridian Health	3. Mayo Clinic (3 locations: Phoenix, AZ, Jacksonville, FL, Rochester MN)
4. Memorial Sloan Kettering Cancer Center	5. Mount Sinai	6. New York University
7. Northwestern University	8. University of California-Davis	9. University of California-San Francisco
10. University of Chicago	11. University of Illinois at Chicago	12. University of Kansas
13. University of Maryland	14. University of Michigan	15. University of North Carolina
16. University of Utah	17. University of Wisconsin	18. Vanderbilt University
19. Virginia Commonwealth University	20. Washington University	21. Yale University

Figure S1. CEC-UW Participating health systems.
The map shows the geographic location of each the 21 health systems. The numbers on the map correspond to the health systems listed in the table below.
